# Foam sclerotherapy for lower-limb varicose veins: impact on saphenous vein diameter

**DOI:** 10.1590/0100-3984.2017.0184

**Published:** 2018

**Authors:** Rodrigo Gomes de Oliveira, Domingos de Morais Filho, Carlos Alberto Engelhorn, Iruena Moraes Kessler, Felipe Coelho Neto

**Affiliations:** 1 Pontifícia Universidade Católica do Paraná (PUC-PR), Curitiba, PR, Brazil.; 2 Universidade Estadual de Londrina (UEL), Londrina, PR, Brazil.; 3 Universidade de Brasília (UnB), Brasília, DF, Brazil.

**Keywords:** Ultrasonography, Varicose veins, Sclerotherapy, Saphenous vein, Venous insufficiency

## Abstract

**Objective:**

To assess changes in the great saphenous vein (GSV) after foam sclerotherapy for varicose veins.

**Materials and Methods:**

This was a prospective study of 33 patients who were treated with polidocanol foam sclerotherapy after having had varicose veins with a clinical severity-etiology-anatomy-pathophysiology classification of C4-C6 for three months. The patients were evaluated by ultrasound before, during, and after the procedure (on post-procedure days 7, 15, 30, 60, and 90). The GSV diameter, the rate of venous occlusion, and the rate of reflux elimination were determined. Two patients were excluded for having a history of deep vein thrombosis history, and one was excluded for having bronchial asthma.

**Results:**

Thirty patients (26 females and 4 males, with mean age of 62 years) completed the protocol. The mean pre-procedure GSV diameter was 6.0 ± 0.32 mm (range, 3.6-11.2 mm). During the sclerotherapy, the mean GSV diameter was reduced to 1.9 ± 0.15 mm (range, 0.6-3.8 mm). On post-procedure day 7, the mean GSV diameter increased to 6.3 ± 0.28 mm (range, 3.9-9.7 mm). On post-procedure day 90, the mean GSV diameter was 4.0 ± 0.22 mm (range, 1.9-8.2 mm). The rate of GSV reflux was significantly lower in the assessment performed on post-procedure day 90 than in the pre-procedure assessment (*p* < 0.0028).

**Conclusion:**

On the basis of our ultrasound analysis, we can conclude that foam sclerotherapy for varicose veins results in a significant reduction in GSV diameter, as well as in the elimination of GSV reflux.

## INTRODUCTION

The ideal treatment for primary varicose veins in the lower extremities should be minimally invasive, repeated when necessary, and free from significant complications. It should also be effective for eliminating points of reflux and reducing venous hypertension at the extremities, as well as being affordable, providing esthetic improvement, and requiring patients to take little time off from work^(^^1^^)^.

The advent of Doppler vascular ultrasound has driven new endovascular treatment methods, such as laser photocoagulation, radiofrequency ablation, and foam sclerotherapy, the last having proven to be an attractive technique, because anesthesia, hospital admission, and post-treatment bed rest are unnecessary^(^^2^^)^. The foam is produced by mixing a sclerosing agent with room air and can be used to treat saphenous veins as well as trunk varicose veins and perforating veins, particularly in advanced cases with skin lesions and ulcers^(^^3^^,^^4^^)^. Doppler vascular ultrasound is indispensable for performing the procedure and for follow up, because it can monitor occlusion of the vein and detect reflux relapses^(^^5^^)^.

The objective of this study was to use duplex ultrasound to evaluate changes in the diameter of the great saphenous vein (GSV) and the rate of venous reflux elimination after treatment with polidocanol foam sclerotherapy.

## MATERIALS AND METHODS

This was an open prospective study evaluating the impact that polidocanol foam sclerotherapy for chronic venous insufficiency (CVI) has on the diameters of the saphenous veins treated and on the rate of venous occlusion. The study was conducted at a private vascular surgery clinic between February and September of 2009. The study was approved by the institutional ethics committee, and all participating patients gave written informed consent.

The inclusion criteria were being ≥ 18 years of age; having lower-limb CVI classified as clinical stage C4, C5, or C6 according to the clinical severity-etiology-anatomy-pathophysiology (CEAP) classification^(^^6^^)^; and proximal, multisegmental, or diffuse reflux in the GSV^(^^7^^)^. Patients in whom ultrasound showed acute deep vein thrombosis or deep vein thrombosis that was not recanalized were excluded, as were those with varicose veins unrelated to GSV reflux and those who had previously undergone varicose vein surgery, as well as those with thrombophilia, active neoplasm or cancer under surveillance, lung disease, or peripheral arterial disease (ankle-brachial index < 0.9).

### Sclerotherapy technique

The sclerotherapy technique employed in the present study has been described elsewhere^(^^8^^)^. The patients underwent treatment of the GSV and of tributary varicose veins. When complete closure of target veins was not achieved, additional sessions were conducted until complete closure was achieved. Additional sessions, with one or more punctures, were conducted, as necessary (on a case-by-case basis), at 7-day intervals. In all cases, foam was produced by mixing 3% polidocanol with room air, at a proportion of 1:4, and the total volume of foam per session did not exceed 10 mL^(^^9^^)^.

After the injection of the foam, the limb was elevated and bound with 12-cm wide inelastic bandages, which were left in place for three days, and patients wore 30/40 mmHg 7/8 elastic compression stockings for three months thereafter. At the end of the procedure, patients were instructed to walk and to resume their normal routines.

### Ultrasound assessment

The examinations were standardized and were all conducted by the same physician. Patients were examined in a standing position with their weight on the contralateral leg, with the limb being examined in external rotation and the calf musculature relaxed, maintaining stability. We employed an ultrasound system (EnVisor; Philips Medical Systems, Andover, MA, USA) with a 10-12 MHz multifrequency transducer. The deep vein system was assessed for acute or previous venous thrombosis. The examination of the superficial vein system focused on the saphenofemoral and saphenopopliteal junctions, together with the great and small saphenous veins, as well as on identifying incompetent perforating veins. Reflux was induced by manual compression of the calf and defined as flow in the retrograde direction for periods greater than 0.5 s for saphenous veins and 0.35 s for perforating veins^(^^10^^)^. The GSV diameter was measured at three points: at the saphenofemoral junction; in the proximal third of the thigh, 5 cm distal to the inguinal crease; in the middle third of the thigh, between the inguinal crease and the knee joint line; and in the distal third of the thigh, 5 cm proximal to the knee joint line. The mean of these measurements was calculated for the purposes of comparison.

In most cases, patients underwent seven ultrasound assessments: the first assessment-performed prior to the procedure, to identify reflux patterns; the second assessment-performed during the procedure, with the objective of guiding puncture of the GSV, monitoring injection of the foam and preventing it from entering the deep vein system; the third assessment-performed on post-procedure day 7, with the objective of detecting deep vein thromboses and verifying the occlusion of the GSV; the fourth, fifth, and sixth assessments-performed on post-procedure days 15, 30, and 60, respectively, to evaluate venous thrombus and changes in the diameter of the GSV; and the seventh assessment-performed on post-procedure day 90, to assess treatment efficacy. Patients in whom the GSV was not occluded by the time of the third assessment underwent another foam sclerotherapy session and an assessment following the criteria and objectives of the second assessment, therefore undergoing a total of eight ultrasound assessments.

### Statistical analysis

The Bartlett test was applied in order to assess the homogeneity of variances, and the Shapiro-Wilk test was employed to determine whether the study data fitted a normal distribution. Fisher's exact test was used in order to determine whether there was an association between treatment failure and occlusion of the GSV. Statistical calculations were made on an electronic spreadsheet (Microsoft Excel 2000), and statistical analyses were performed with the IBM SPSS Statistics software package, version 23.0 (IBM Corporation, Armonk, NY, USA).

## RESULTS

We recruited 33 patients with primary varicose veins in the lower extremities. In all of the patients, the primary varicose veins were classified as CEAP clinical stage C4, C5, or C6 and GSV reflux was classified as proximal, multisegmental, or diffuse. Of those 33 patients, 3 were excluded: two for having a history of deep vein thrombosis, and 1 for having bronchial asthma. Therefore, a total of 30 patients-26 women and 4 men, with a mean age of 62 years (range, 32-70 years)-completed the 90-day protocol. 

The primary varicose vein was classified as CEAP C4 in 19 patients (63.3%), as CEAP C5 (with healed ulcers) in 5 (16.7%), and as CEAP C6 (with unhealed ulcers) in 6 (20.0%). All of the patients showed GSV reflux, which was classified as diffuse in 12 patients (40%) and as proximal in 9 (30%). In the pre-procedure assessments, the mean GSV diameter was 6.0 ± 3.2 mm (range, 3.6-11.2 mm).

**Figure 1:**
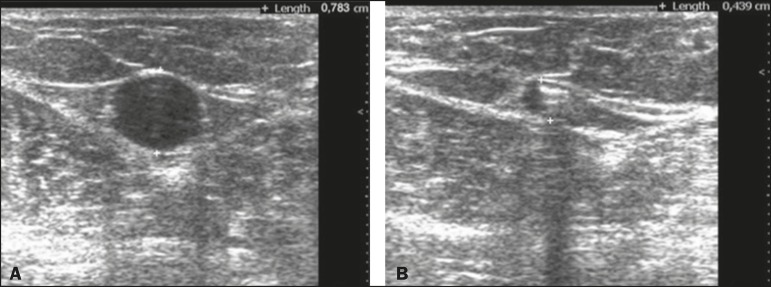
Ultrasound images of the GSV. **A:** Caliber of the vein before treatment (d = 7.8 mm). **B:** Reduction in caliber in response to injection of the foam (d = 4.3 mm).

The first change to the saphenous vein observed on duplex ultrasound immediately after injection of the foam is a rapid reduction in the caliber of the vessel, caused by the vasospasm provoked by the foam. Figure 1 illustrates the reduction in caliber of the saphenous vein in one of the patients evaluated in the present study. During sclerotherapy, the mean GSV diameter was 1.9 ± 0.15 mm (range, 0.6-3.8 mm). In the follow-up ultrasound examination performed on post-procedure day 7, the calibers of treated GSVs had increased, the mean diameter being 6.3 ± 0.28 mm (range, 3.9-9.7 mm). All veins that had been treated with sclerotherapy were not compressible with the transducer, exhibiting parietal thickening, as well as luminal content with a homogenous appearance that was predominantly hypoechoic. In all cases, there was no flow seen on color or pulsed-wave Doppler.

**Figure 2:**
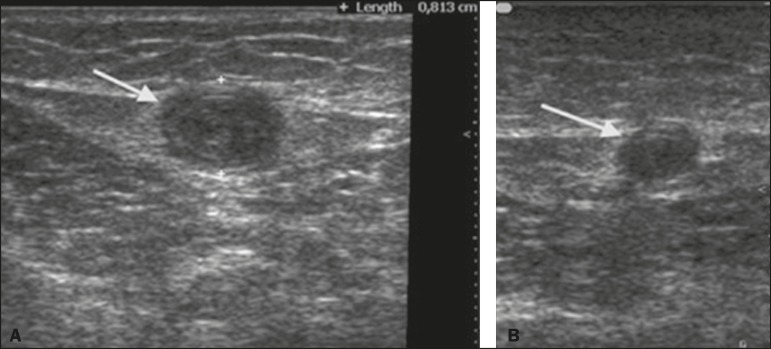
Ultrasound images of the GSV after the procedure. **A:** Caliber of the vein at 7 days after the procedure (d = 8.2 mm). **B:** Caliber of the vein at 90 days after the procedure (d = 5.1 mm).

To achieve total occlusion of the GSV, 26 patients (86.6%) required only a single session of sclerotherapy, 3 (10%) required two sessions, and 1 (3.3%) required three sessions. During the subsequent examinations, gradual contraction of the luminal content was observed and, consequently, there was a reduction in the mean diameter of the saphenous veins treated. The mean GSV diameter was 5.7 mm (range, 3.3-9.8 mm) on post-procedure day 15, 5.0 mm (range, 3.1-9.2 mm) on post-procedure day 30, 4.4 mm (range, 2.3-8.8 mm) on post-procedure day 60, and 4.0 mm (range, 1.9-8.2 mm) on post-procedure day 90. There was a statistically significant difference between the mean GSV diameter on post-procedure day 90 and that recorded at baseline (*p* < 0.001). Figure 2 illustrates the GSV diameter on post-procedure days 7 and 90.

**Figure 3:**
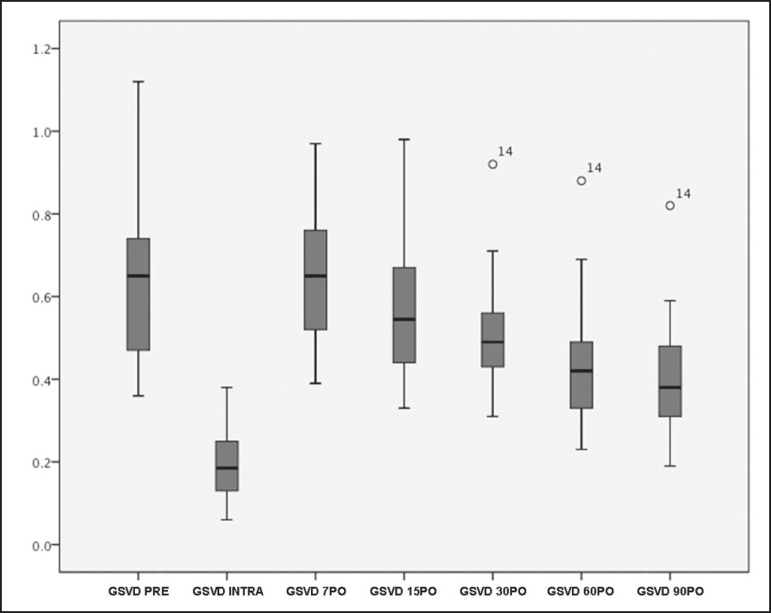
Measurements of GSV diameter before, during, and after procedure, up to post-procedure day 90.

Figure 3 illustrates the evolution of the mean GSV diameter over time. Before treatment, the mean diameter was 6.0 mm, decreasing to 1.9 mm immediately after injection of the foam. By post-procedure day 7 days, the mean GSV diameter had increased to 6.3 mm and underwent a gradual reduction over the course of the subsequent assessments, reaching 4.0 mm at 90 days after the treatment.

Duplex ultrasound analysis revealed a statistically significant reduction in the proportion of patients showing GSV reflux, from the pre-procedure rate of 100% to a 90-day post-procedure rate of 13.3% (*p* = 0.0028). In terms of the elimination of reflux, 22 (73%) of the saphenous veins treated were found to be totally occluded on post-procedure day 90. At that same time point, we identified eight recanalizations, of which seven were partial (three with reflux and four without) and just one was complete (with reflux).

With regard to complications, eight (27%) of the patients experienced episodes of scintillating scotoma after injection of the foam, without any other systemic complications. No major events, such as deep venous thrombosis or pulmonary embolism, were observed.

## DISCUSSION

Successful sclerotherapy for the treatment of lower-extremity varicose veins requires detailed planning. In general, sclerotherapy is conducted respecting the order of the reflux points, working from larger caliber varicose veins to those with smaller caliber^(^^11^^)^. Therefore, adequate clinical and anatomic assessments must be performed before treatment^(^^12^^)^.

In addition to the clinical assessment, vascular ultrasound plays a crucial role in the assessment of venous reflux in the lower extremities. It is a low-cost, noninvasive examination that is well tolerated by patients. It offers direct visualization, localization, and quantification of venous reflux with 95% sensitivity and 100% specificity^(^^10^^)^.

This study assessed the impact that CVI treatment with foam sclerotherapy had on the GSV diameter and the rate of venous occlusion. The majority of the patients in the sample were classified as being in the more advanced stages of the disease, and all the patients showed GSV reflux.

The fact that there were no major complications, such as deep venous thrombosis or pulmonary embolism, in our sample is in line with other findings in the medical literature^(^^13^^)^. The pigmentation rate in our sample was 13%, phlebitis requiring drainage occurring in two cases (6%). In one case, we observed partial propagation of the thrombus from the GSV into the deep vein system, although it affected less than 50% of the femoral vein lumen, with spontaneous regression and no need for treatment. In keeping with the results of one systematic review^(^^13^^)^, we observed post-procedure phlebitis in 16 patients (53%), although drainage of clots was necessary in only two of those patients.

Reduction in the caliber of treated veins is a common finding in post-procedure ultrasound examinations^(^^14^^)^. In our patient sample, we observed a statistically significant (33%) reduction in GSV diameters at 90 days after sclerotherapy.

Rasmussen et al.^(^^15^^)^ compared four techniques available for treatment of saphenous vein reflux (intravenous laser ablation, radiofrequency ablation, foam sclerotherapy, and conventional surgery) and observed that the recanalization rate after foam sclerotherapy was approximately 20%, without repercussions for symptoms, as reflected in the results on quality-of-life questionnaires. In the present study, the rate of reflux elimination at 90 days was 87%, which is comparable to rates described in the literature^(^^13^^)^. Analysis of the rates of saphenous vein occlusion shows that the results of foam sclerotherapy are systematically inferior to those of thermal ablation techniques and conventional surgery^(^^14^^-^^16^^)^. Nevertheless, the lower rates of occlusion and higher rates of recanalization/reflux relapse in veins treated with foam do not result in worse patient quality-of-life scores^(^^14^^-^^17^^)^. One possible explanation for the maintenance of quality-of-life scores, even after recanalization with reflux in treated saphenous veins, is the considerable reduction in vein diameter and the closure of tributaries that drain reflux.

Although our study has some limitations, including the small sample size and relatively short follow-up period, it has demonstrated that foam sclerotherapy effectively eliminated axial reflux in saphenous veins and resulted in a significant reduction in venous diameters. Further studies are needed in order to determine whether the reduction of venous diameter truly contributes to the maintenance of the good quality-of-life scores obtained with foam sclerotherapy for CVI.
